# Epigenetic clocks and gliomas: unveiling the molecular interactions between aging and tumor development

**DOI:** 10.3389/fmolb.2024.1446428

**Published:** 2024-07-26

**Authors:** Shiliang Chen, Yi Jiang, Cong Wang, Shiyuan Tong, Yibo He, Wenqiang Lu, Zhezhong Zhang

**Affiliations:** ^1^ Department of Clinical Lab, The First Affiliated Hospital of Zhejiang Chinese Medical University (Zhejiang Provincial Hospital of Chinese Medicine), Hangzhou, China; ^2^ Department of Intensive Care Unit, Suzhou Kowloon Hospital, Shanghai Jiao Tong University School of Medicine, Suzhou, Jiangsu, China; ^3^ State Key Laboratory of Medical Neurobiology and MOE Frontiers Center for Brain Science, Institutes of Brain Science, Fudan University, Shanghai, China; ^4^ Department of Thoracic Surgery, Suzhou Kowloon Hospital, Shanghai Jiao Tong University School of Medicine, Suzhou, Jiangsu, China

**Keywords:** glioma, epigenetic clock, DNA methylation, aging, personalized medicine

## Abstract

Gliomas, the most prevalent and aggressive primary brain tumors, represent a diverse group of malignancies originating from glial cells. These tumors account for significant brain tumor-related morbidity and mortality, with higher incidence rates in North America and Europe compared to Asia and Africa. Genetic predispositions and environmental factors, particularly ionizing radiation, critically impact glioma risk. Epigenetics, particularly DNA methylation, plays a pivotal role in glioma research, with IDH-mutant gliomas showing aberrant methylation patterns contributing to tumorigenesis. Epigenetic clocks, biomarkers based on DNA methylation patterns predicting biological age, have revealed significant insights into aging and tumor development. Recent studies demonstrate accelerated epigenetic aging in gliomas, correlating with increased cancer risk and poorer outcomes. This review explores the mechanisms of epigenetic clocks, their biological significance, and their application in glioma research. Furthermore, the clinical implications of epigenetic clocks in diagnosing, prognosticating, and treating gliomas are discussed. The integration of epigenetic clock data into personalized medicine approaches holds promise for enhancing therapeutic strategies and patient outcomes in glioma treatment.

## Introduction

Gliomas are the most prevalent and aggressive primary brain tumors, comprising a diverse group of malignancies that originate from glial cells in the central nervous system (Hajianfar et al., 2023). These tumors represent about one-third of all primary brain and central nervous system (CNS) tumors and are responsible for a significant proportion of brain tumor-related morbidity and mortality. The incidence of gliomas varies globally, with higher rates observed in North America and Europe compared to Asia and Africa. Several studies highlight the critical impact of genetic predispositions and environmental factors, such as exposure to ionizing radiation, on glioma risk (Ostrom et al., 2015; Cahill and Turcan, 2018; Stabellini et al., 2021). Glioblastoma multiforme (GBM) is the most common and lethal subtype, characterized by its resistance to conventional therapies and poor prognosis, with median survival times of approximately 15 months despite treatment advancements (Ostrom et al., 2018; Grochans et al., 2022). Gliomas, particularly GBM, present significant challenges in neuro-oncology due to their aggressive nature and poor prognosis (Romano et al., 2022). Research hotspots in glioma include the exploration of genetic mutations, tumor microenvironment, and resistance to conventional therapies. The treatment and prognosis of gliomas have been significantly influenced by genetic and epigenetic factors. Molecular diagnostics, such as the detection of IDH mutations, MGMT methylation status, and 1p/19q co-deletion, play critical roles in guiding treatment strategies and predicting patient outcomes (Ho et al., 2014).

Epigenetics refers to the study of heritable changes in gene expression that do not involve changes to the underlying DNA sequence. These changes are crucial in regulating gene activity and can be influenced by various factors including environment, lifestyle, and disease states (Smith and Doe, 2019). A key epigenetic mechanism is DNA methylation, which involves the addition of a methyl group to the 5-carbon of the cytosine, often resulting in gene silencing (Brown and Green, 2021). In mammals, DNA methylation predominantly occurs at CpG dinucleotide sequences, with the number of CpG sites exceeding 28 million (Stirzaker et al., 2014). The abundance of CpG sites provides a crucial foundation for the specific epigenetic silencing of genes. Numerous studies have demonstrated the significant role of epigenetics in cancer development. Aberrant DNA methylation patterns, such as hypermethylation of tumor suppressor genes and global hypomethylation, are commonly observed in various cancers. In gliomas, especially those with isocitrate dehydrogenase (IDH) mutations, abnormal DNA methylation patterns are frequently observed. These aberrations can lead to dysregulated gene expression and contribute to tumor development and progression (Molinaro et al., 2019). In the realm of epigenetics, understanding how epigenetic modifications like DNA methylation, histone modification (Wang et al., 2022), and non-coding RNA regulation contribute to tumorigenesis and therapy resistance is crucial. Challenges in glioma research include the heterogeneity of gliomas, which complicates the development of universal treatment strategies, and the difficulty in achieving long-term therapeutic success due to the tumor’s adaptive mechanisms. Epigenetics offers a promising avenue for addressing these challenges by revealing mechanisms of gene regulation that do not involve changes to the DNA sequence but affect gene expression and cellular behavior.

**FIGURE 1:**
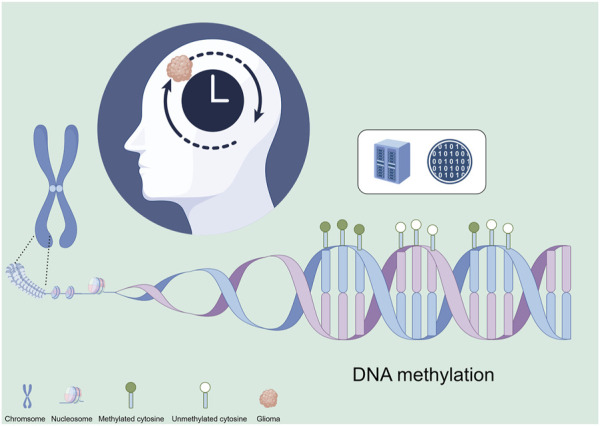
Epigenetic clock construction for gliomas based on epigenetics. This figure was created using the Figdraw online drawing tool.

DNA methylation patterns are tissue-specific and vary significantly across different cell types. These patterns also correlate strongly with age. Studies have shown that as individuals age, there is a global decrease in DNA methylation (hypomethylation) alongside site-specific increases (hypermethylation). These changes can lead to altered gene expression and a decline in cellular function, contributing to aging and age-related diseases (Johnson et al., 2020). Epigenetic clocks are biomarkers based on DNA methylation patterns that can predict biological age and have been linked to aging and various diseases, including cancers (Figure 1). These clocks measure the cumulative effect of an individual’s epigenetic maintenance system and have been used to understand the relationship between aging and tumor development. Recent studies have demonstrated that the acceleration of epigenetic aging is associated with increased cancer risk and poorer outcomes. In gliomas, research on epigenetic clocks has revealed significant insights into the timing of tumorigenesis and potential therapeutic targets (Gieryng et al., 2017).

## Epigenetic clocks—Mechanisms and biological significance

The epigenetic clock refers to a biological timing method based on DNA methylation patterns, used to estimate the physiological age of an organism. This approach utilizes changes in the methylation status of CpG sites in specific genomic regions to infer an individual’s age. The epigenetic clock is a powerful tool, offering a new dimension for evaluating and understanding the aging process by revealing the patterns of DNA methylation changes associated with age. The two most widely recognized models are Horvath’s clock and Hannum’s clock. Horvath’s clock employs 353 CpG sites to estimate the biological age of various human tissues and cell types (Horvath, 2013), whereas the Hannum clock focuses on blood-specific CpG sites (Armstrong et al., 2017). The construction of these clocks typically involves elastic net regression modeling, which helps in selecting CpG sites that contribute significantly to age prediction while avoiding overfitting (Liu et al., 2019). These models have significantly advanced our understanding of aging and its impact on health and disease. This method has been shown to achieve high accuracy, with correlation coefficients often exceeding 0.95 between predicted and chronological age in various tissue types (Liu et al., 2020). Integrating the strengths of these models, such as Horvath’s broader tissue applicability and Hannum’s specificity to blood, can lead to the development of more precise and practical composite models. These integrated models can enhance the accuracy and efficacy of biological age prediction and improve our understanding of cancer biology and aging.

Epigenetic clocks are invaluable in reflecting cellular aging and have been associated with various age-related diseases. The application of epigenetic clocks involves comparing the methylation levels at these key sites to reference data from healthy individuals, allowing researchers to estimate the biological age of tissues (Horvath and Raj, 2018). Accelerated epigenetic aging, as indicated by a higher predicted biological age compared to chronological age, has been linked to increased risk of multiple diseases including cancer, cardiovascular diseases, and neurodegenerative disorders. For example, epigenetic age acceleration is significantly associated with Alzheimer’s disease and related neuropathologies (Grodstein et al., 2021). Research has demonstrated that epigenetic clocks are robust across various applications and tissues. For instance, the GrimAge clock has shown superior predictive power for age-related clinical phenotypes and all-cause mortality compared to other models (McCrory et al., 2020). Furthermore, these clocks are being increasingly utilized in understanding the impact of environmental factors (Yang et al., 2023) on aging. Studies have found significant associations between socioeconomic status, environmental exposures, and epigenetic age acceleration, suggesting that these clocks can also serve as indicators of life course socio-environmental stress (Lawrence et al., 2020). In cancer research, studies have shown that patients with higher epigenetic age acceleration are more likely to develop malignancies and experience poorer outcomes. In gliomas, accelerated epigenetic aging is correlated with more aggressive tumor phenotypes and worse survival rates, underscoring the potential of epigenetic clocks as prognostic biomarkers (Romani et al., 2018).

## Epigenetic alterations in gliomas

Epigenetic alterations, including DNA methylation, histone modifications, and non-coding RNAs, play significant roles in glioma biology. These modifications affect gene expression and chromatin structure, contributing to the initiation and progression of gliomas. Epigenetic modifications, particularly DNA methylation and histone modification, play significant roles in the pathogenesis of gliomas.

### DNA methylation in gliomas

DNA methylation is catalyzed by DNA methyltransferases (DNMTs), while demethylation involves the action of ten-eleven translocation (TET) enzymes that oxidize 5-methylcytosine to 5-hydroxymethylcytosine, leading to subsequent removal of the methyl group (Gusyatiner and Hegi, 2018). Gliomas often exhibit aberrant DNA methylation, which can lead to the silencing of tumor suppressor genes and other critical regulatory genes. The interplay between genetic mutations and epigenetic modifications is crucial in glioma pathogenesis. Mutations in the IDH genes lead to a specific hypermethylation phenotype known as the glioma CpG island methylator phenotype (G-CIMP), which is associated with better prognosis and survival (Christensen et al., 2011). The G-CIMP phenotype results from IDH mutations leading to global DNA hypermethylation, affecting gene expression and tumor behavior (LeBlanc and Marra, 2016). Research has identified several DNA methylation patterns associated with gliomas, particularly within the promoter regions of genes involved in cell cycle regulation, apoptosis, and DNA repair (Weng and Salazar, 2021). For instance, aberrant methylation of the MGMT promoter is a well-documented marker, linked to better responses to alkylating agents like temozolomide and improved survival rates (Aoki and Natsume, 2019). Additionally, DNA methylation patterns can stratify gliomas into distinct prognostic subgroups, with certain methylation markers predicting poor survival outcomes (Chen et al., 2019).

### Histone modifications in gliomas

Histone modifications, such as acetylation, methylation, phosphorylation, and ubiquitination, regulate chromatin structure and gene expression. These modifications occur on the N-terminal tails of histones and can either activate or repress transcription. In gliomas, alterations in histone-modifying enzymes are common and contribute to oncogenesis (Dabrowski and Wojtas, 2019), such as histone methyltransferases and deacetylases. For example, mutations in histone H3 variants (H3.1 and H3.3) are associated with pediatric high-grade gliomas and result in altered global H3K27me3 levels, impacting gene expression and cell fate (Maleszewska and Kamińska, 2015). Altered expression of histone-modifying enzymes, such as EZH2 (a histone methyltransferase), can lead to changes in chromatin structure that promote glioma cell proliferation and survival.

### Non-coding RNAs in gliomas

Non-coding RNAs, including microRNAs (miRNAs) and long non-coding RNAs (lncRNAs), play crucial roles in regulating gene expression at the post-transcriptional level. miRNAs, typically 20-22 nucleotides long, can bind to complementary sequences in mRNAs, leading to mRNA degradation or inhibition of translation. In gliomas, dysregulation of specific miRNAs and lncRNAs contributes to tumorigenesis by modulating pathways involved in cell proliferation, apoptosis, and migration. For example, miR-185 targets DNA methyltransferases and regulates global DNA methylation in gliomas (Zhang et al., 2011). For instance, the lncRNA MEG3 is often silenced in gliomas due to promoter hypermethylation, leading to the repression of the p53 pathway and promoting tumor growth (Li et al., 2016). Dysregulation of miRNAs, such as miR-21, and lncRNAs, such as HOTAIR, can affect multiple signaling pathways involved in glioma pathogenesis, including those regulating cell cycle and apoptosis.

## Connections between epigenetic clocks, aging, and gliomas

### Intersection of gliomas and aging

Aging is a well-known risk factor for many cancers, including gliomas. Epigenetic changes, such as DNA methylation, play a crucial role in both aging and cancer (Zheng et al., 2024). As individuals age, global DNA hypomethylation and site-specific hypermethylation occur, affecting gene expression and genomic stability. This process can contribute to the development of gliomas by promoting genetic mutations and altering cellular processes (Castro and McClellan, 2023). Studies have shown that gliomas exhibit accelerated epigenetic aging, which is characterized by the disparity between an individual’s chronological age and their biological age as predicted by epigenetic clocks (Liao et al., 2018; Arrieta et al., 2023).

### Application of epigenetic clocks in glioma research

Epigenetic clocks, such as Horvath’s clock and epiTOC, have been employed to study aging in gliomas. These clocks measure DNA methylation patterns at specific CpG sites to estimate biological age. Research has demonstrated that gliomas, especially high-grade gliomas, exhibit accelerated epigenetic aging compared to normal brain tissues (Bady et al., 2022; Dou et al., 2023). This accelerated aging is associated with poor prognosis and increased tumor aggressiveness. For example, studies have found that the epigenetic age of gliomas is often significantly higher than the chronological age of patients, suggesting a link between epigenetic dysregulation and tumor development (Zheng et al., 2020). As individuals age, DNA methylation patterns undergo significant changes, typically characterized by global hypomethylation and locus-specific hypermethylation. These changes affect gene expression profiles and contribute to age-related decline in cellular function. Epigenetic clocks capture these methylation alterations, reflecting the biological aging process at the molecular level. For instance, the hypermethylation of tumor suppressor genes and hypomethylation of oncogenes are common in aging tissues and are indicative of age-related diseases, including cancer (Liao et al., 2018). Epigenetic clocks show varying performance across different glioma types. For example, IDH-mutant gliomas often exhibit distinct DNA methylation patterns, leading to better prognostic outcomes compared to IDH-wildtype gliomas. These differences highlight the importance of considering tumor-specific epigenetic profiles when using epigenetic clocks for prognosis and treatment planning.

### Mechanisms of accelerated epigenetic aging in gliomas

The mechanisms by which gliomas affect epigenetic clock readings are complex and multifactorial. Gliomas can alter the epigenetic landscape through mutations in genes involved in DNA methylation and histone modification. For instance, mutations in the IDH1 and IDH2 genes produce 2-hydroxyglutarate, which inhibits TET enzymes and leads to DNA hypermethylation. Additionally, the disruption of chromatin remodelers and histone modifiers can further exacerbate epigenetic dysregulation. These changes contribute to the observed accelerated epigenetic aging in glioma tissues (Romani et al., 2018).

### Studies on accelerated epigenetic aging in gliomas

Multiple studies have confirmed the presence of accelerated epigenetic aging in gliomas. Early research by Natsume et al. (2010) focused on DNA methylation and CpG island hypermethylation in gliomas, highlighting their role in treatment response and prognosis. Kreth et al. (2014) reviewed epigenetic mechanisms in gliomas, discussing their diagnostic and therapeutic potential. Bai et al. (2016) provided insights into the genetic and epigenetic mechanisms driving glioma progression. Klughammer et al. (2017) mapped DNA methylation in primary and recurrent glioblastomas, revealing epigenetic changes during progression. Liao et al. (2018) found that the epigenetic ages of gliomas calculated using Horvath’s clock and epiTOC were significantly higher than those of normal brain tissues. This acceleration was consistent across different glioma subtypes and was associated with worse clinical outcomes. Similarly, Zheng et al. (2020) showed that epigenetic age acceleration could serve as a prognostic biomarker for glioma patients, with higher acceleration correlating with reduced survival rates. Epigenetic clock data can identify specific methylation changes in gliomas, facilitating the development of targeted therapies for these alterations. For instance, DNMT inhibitors can be employed to reverse abnormal DNA methylation patterns in patients with epigenetic abnormalities (Rajendran et al., 2011).

## Clinical significance of epigenetic clocks in gliomas

### Diagnostic and prognostic potential

Research on epigenetic clocks has demonstrated their potential as both diagnostic and prognostic markers for gliomas. Epigenetic clocks, which utilize DNA methylation patterns to estimate biological age, have been shown to predict glioma outcomes. For example, Horvath’s clock and epiTOC have been used to assess epigenetic aging in gliomas, revealing that these tumors often exhibit accelerated epigenetic aging compared to normal brain tissue. This accelerated aging is associated with poorer prognosis and more aggressive tumor behavior. Epigenetic clocks have shown promise in the early diagnosis of gliomas. Integrating epigenetic clock data into clinical practice offers numerous benefits, such as early detection of gliomas through non-invasive blood tests and improved prognostic assessments. For instance, the detection of tumor-specific DNA methylation patterns in circulating cell-free DNA (cfDNA) from blood samples enables earlier detection and better monitoring of tumor progression (Souza et al., 2018; Cao et al., 2023). Genome-wide DNA methylation profiling in liquid biopsies has enabled the identification of specific epigenetic signatures that accurately predict glioma presence and distinguish between prognostic subtypes (Lee et al., 2019). Addition ally, circadian clock genes, which are regulated by epigenetic mechanisms, have been identified as independent prognostic markers, providing further insight into glioma biology and patient outcomes (Chai et al., 2022). For example, CpG sites associated with glioma grade and specific molecular alterations can predict patient survival, underscoring the biological significance of epigenetic modifications in glioma progression (Weng and Salazar, 2021). Accelerated epigenetic senescence, indicated by higher epigenetic age acceleration, correlates with poor overall survival and increased tumor invasiveness. Patients with higher epigenetic age acceleration exhibit significantly reduced survival rates compared to those with lower acceleration, supporting the prognostic value of epigenetic clocks in clinical practice (Grodstein et al., 2021).

### Impact on treatment

Targeting epigenetic mechanisms in glioma treatment represents a promising therapeutic strategy. DNMT inhibitors and HDAC inhibitors (Duan et al., 2023) are examples of epigenetic therapies that aim to reverse abnormal DNA methylation and histone modification patterns observed in gliomas. These therapies have the potential to modulate the biological age of glioma cells, thereby affecting tumor behavior and improving patient outcomes. Epigenetic therapy holds significant promise for glioma treatment. DNMT inhibitors (e.g., decitabine and azacitidine) can reverse abnormal DNA methylation patterns, reactivating silenced tumor suppressor genes and sensitizing tumors to conventional therapies (Romani et al., 2018). HDAC inhibitors (e.g., vorinostat and panobinostat) modulate chromatin structure and gene expression by inhibiting histone deacetylases, which can reduce tumor growth and induce apoptosis in glioma cells (Faria et al., 2020). Additionally, integrating epigenetic clock data into personalized medicine approaches can enhance treatment plans by tailoring therapies based on the epigenetic profile of individual tumors (Liu et al., 2022; Chen et al., 2023; Lv et al., 2023; Griñán-Ferré et al., 2024).

Personalized medicine approaches that incorporate epigenetic clock data are becoming increasingly feasible (Li et al., 2023; Xiong et al., 2023). By integrating epigenetic information, clinicians can develop individualized treatment plans that consider the specific epigenetic alterations present in a patient’s tumor (Shen et al., 2018). This approach not only improves the accuracy of prognosis but also helps in predicting treatment response and tailoring therapeutic interventions accordingly (Han et al., 2020; Polano et al., 2021). However, challenges such as the need for robust and standardized epigenetic assays and the integration of complex epigenetic data into clinical workflows remain (Jones et al., 2019). Several challenges arise in clinical practice, including the standardization of techniques, ensuring consistency in DNA methylation measurements across different laboratories, and the interpretation of complex epigenetic data. Addressing these challenges requires developing standardized protocols for sample collection, processing, and analysis. Additionally, establishing comprehensive databases and bioinformatics tools for data interpretation can aid clinicians in making informed decisions. Ethical issues such as privacy concerns and informed consent must also be addressed by implementing robust data protection measures and ensuring transparent communication with patients about the use and implications of their epigenetic data.

## Future directions and research opportunities

One significant unsolved problem in glioma research is the gap in understanding the relationship between the epigenetic clock and glioma development and progression. Epigenetic clocks, which measure biological aging through DNA methylation patterns, have shown potential as biomarkers for cancer prognosis but their specific role in gliomas remains unclear (Liao et al., 2018). Moreover, longitudinal studies tracking epigenetic changes in glioma patients over time are necessary to elucidate the dynamics of epigenetic modifications and their impact on tumor behavior and patient outcomes (De La Cruz Minyety et al., 2021). Furthermore, large-scale, multicenter studies are essential to validate the clinical application of epigenetic clocks in diagnosing and prognosticating gliomas (Morales La Madrid et al., 2015). Multicenter studies are crucial for validating the stability and applicability of epigenetic clocks across diverse patient populations and experimental conditions. Integrating data from different centers helps to identify and eliminate potential biases and confounding factors, leading to more comprehensive and objective conclusions.

Advanced technologies such as single-cell sequencing and CRISPR-based epigenome editing hold great promise for studying epigenetic changes in gliomas (Romani et al., 2018). Single-cell sequencing allows for the detailed characterization of the epigenetic landscape at a cellular level, enabling the identification of heterogeneity within tumors and the discovery of new therapeutic targets (Filbin and Suvà, 2016). CRISPR-based technologies can be used to edit specific epigenetic marks, providing a powerful tool for functional studies and the development of targeted therapies (Rahme et al., 2023). Epigenetic editing technologies, such as CRISPR-Cas9, offer precise control over DNA methylation and histone modifications. These technologies can target specific epigenetic marks and modulate gene expression with high specificity, potentially leading to more effective and personalized treatment strategies. For instance, CRISPR-Cas9 can be used to demethylate tumor suppressor genes or methylate oncogenes, thereby directly altering the epigenetic landscape of gliomas (Rahme et al., 2023). Current challenges in epigenetic therapy include developing tools that specifically target epigenetic marks without affecting other genomic regions, ensuring efficient and targeted delivery of epigenetic drugs and editing tools to tumor cells, and understanding and overcoming resistance mechanisms that may arise through compensatory pathways or genetic mutations. Concrete research ideas and strategies to address these challenges include investigating the synergistic effects of combining DNMT or HDAC inhibitors with conventional therapies such as chemotherapy and radiotherapy, identifying epigenetic biomarkers that predict response to epigenetic therapies to enable personalized treatment approaches, and conducting longitudinal studies to monitor changes in epigenetic marks and assess the long-term efficacy and safety of epigenetic therapies.

Furthermore, establishing a comprehensive epigenetic database for glioma research, integrating data on histone modifications, DNA mutations, and transcriptome data, can facilitate multi-omics approaches to explore the epigenetic clock in glioma more comprehensively (Polano et al., 2021). This integration will enable researchers to identify novel epigenetic biomarkers and therapeutic targets, potentially leading to more effective and personalized treatments for glioma patients (Castro and McClellan, 2023; McClellan et al., 2023). The application of machine learning and bioinformatics tools further facilitates the integration and interpretation of multi-omics data, leading to the identification of novel biomarkers and therapeutic targets (Shaw et al., 2022). Such collaborative projects could also enhance the development of predictive models and personalized treatment strategies (Reifenberger et al., 2017).

## Conclusion

Epigenetic clocks, measuring biological age via DNA methylation patterns, show accelerated aging in gliomas compared to normal brain tissue, correlating with aggressive behavior and poor prognosis. Specific methylation patterns linked to IDH mutations shape gliomas’ epigenetic landscape. These clocks serve as prognostic biomarkers, predicting outcomes based on epigenetic age acceleration. They aid in early glioma detection and provide prognostic information for accurate disease progression prediction and patient stratification for therapies. Integrating epigenetic clock data into personalized treatment plans enhances therapy effectiveness by tailoring interventions to individual profiles, optimizing responses, and minimizing adverse effects. DNMT and HDAC inhibitors targeting epigenetic alterations hold potential for reversing abnormal changes in gliomas, improving outcomes.
